# Supplementary material for: Are carbon emissions trading and green financial instruments synergistic? -Comprehensive quantitative research based on content analysis
Source: PLoS One. 2024 Mar 7;19(3):e0298601. doi: 10.1371/journal.pone.0298601 (PMC10919601; doi:10.1371/journal.pone.0298601)
Supplement: S1 Data — (ZIP) [file pone.0298601.s001.zip › Data/地级市Z_F工作报告.zip!/║╙─╧/╨φ▓2╩╨/└┤╫╘╣1⁄2╓┌║┼í╛┬φ┐╦╩2╛▌═°í┐_2009.docx]

各位代表：

现在，我代表市人民政府，向大会作工作报告，请予审议，并请市政协各位委员提出意见。

一、2008年政府工作回顾

2008年，是极不平凡的一年。我市经济社会发展经受住了重大的挑战和考验。在省委、省政府和市委的正确领导下，全市上下齐心协力，迎难而上，积极应对不懈怠，科学发展不动摇，较好地完成了市五届人大三次会议确定的各项任务，保持了经济社会平稳较快发展的良好势头。

——经济发展再上台阶，基础更加坚实。全市生产总值突破千亿元大关，达到1062亿元，增长12.6%，一、二、三产业增加值分别增长4.2%、14.9%和10.5%；地方财政一般预算收入41.6亿元，增长23.2%；市本级财政一般预算收入10.3亿元，增长25.7%；全社会固定资产投资524.7亿元，增长32.1%。“1415”主要经济指标顺利实现。

——重点项目加速推进，支撑更加有力。110个重点项目完成投资170亿元。禹州电厂二期工程、许昌铁矿、石武铁路客专许昌段等工业和交通项目相继开工建设，东城区热电厂、烟草薄片生产线等项目竣工投产。中原电气谷核心区、帝豪品牌卷烟专线等重大前期项目取得突破性进展。

——发展质量显著提高，步伐更加稳健。全市规模以上工业实现利润194亿元，增长25%。税收占地方财政一般预算收入的比重达到78.4%。金融机构各项存款余额523亿元，增长19%；贷款余额378.7亿元，增长15.8%。科技进步对经济增长的贡献率达到52%。

——人民生活不断改善，成果更惠民众。“十大实事”全部完成，全市财政用于民生支出39亿元，占一般预算支出的50.9%，增长32.6%。城镇居民人均可支配收入12448元，农民人均纯收入5841元，分别实际增长10%和8.3%。社会消费品零售总额248.6亿元，增长23.8%。

一年来，我们主要做了以下重点工作：

（一）倾力调节经济运行。认真落实中央宏观调控政策，针对年初物价过快上涨，实施临时价格干预措施，有效平抑了市场物价。针对年中生产要素供应偏紧，加强煤电油气运调度，多次组织银企洽谈，保持了工业经济正常运行。针对第三季度以来的严峻形势，制定出台了一系列扩内需、保增长的具体措施，在中央1000亿元新增投资中，争取项目47个、资金4.22亿元，加强了薄弱环节建设，促进了经济持续增长。

（二）注重优化经济结构。着力推动现代产业体系建设，装备制造、能源、食品3大主导产业加快发展，电力电子、超硬材料、发制品、烟草4大特色产业不断壮大，8个工业集聚区列入省重点产业集聚区，“一带十区二十个产业集群”加快发展，正在成为新的增长点。加快发展现代服务业，丹尼斯百货、大商新玛特等大型商贸企业落户许昌，成功举办了第八届中原花木交易博览会和第二届三国文化周，全年接待国内外游客365万人次，增长42%。切实增强自主创新能力，新组建省级工程技术研究中心3个，连续3次获得“全国科技进步先进市”。着力推进节能减排，单位生产总值能耗下降4.13%，化学需氧量、二氧化硫排放量分别削减5321吨和13867吨。

（三）着力解决“三农”问题。继续稳定粮食生产，总产达到270.7万吨，连续5年创历史新高。大力发展现代农业，花木、蔬菜、中药材种植面积分别达到75万亩、100万亩和40万亩；畜牧业完成产值94.5亿元，占农业总产值的45.1%。不断改善农村生产生活条件，完成县乡公路改造321.4公里、通村公路241公里，新解决16.94万人的饮水安全问题，新建农村户用沼气池6.09万口，新增农村连锁超市300个。城乡一体化推进区169个项目完成投资59.8亿元，基础设施不断改善，产业支撑更加有力，生态建设特色鲜明，社会事业统筹发展，创业就业服务体系逐步完善，示范带动作用进一步增强。农村综合改革全面推进，服务体系、集体林权制度改革和土地流转扎实推进，富有实效。

（四）加大城市建设力度。55个城建重点项目完成投资9.36亿元，城镇化率达到37.5%。许由路东段、议台路、清虚街、光明路、向阳路等8条断头路全部贯通。市群众艺术馆、社会福利院、残疾人康复中心、许扶运河游园、南湖游园等建成投用，市文博馆、会展中心和职业技术学院体育馆等开工建设，7个城中村改造项目全面启动。瑞贝卡污水处理厂二期工程、许昌县尚集污水处理厂等城市污水处理项目加快推进。“四城同创”成效显著，成功摘取“国家卫生城市”桂冠，荣获“全国创建文明城市工作先进城市”称号。生态环境进一步改善，空气质量优良天数达到334天。城市管理精细化、数字化和夜景灯饰化工作深入推进，城市品位进一步提升。

（五）继续深化改革开放。许继集团与中国平安实现战略重组，探索了“产融结合”新模式。天健热电与平禹煤电战略重组，青山金汇与广东广新实现合作，28家企事业单位改革改制全面完成，部分改制企业的遗留问题妥善解决。文化体制改革进展顺利，医疗卫生体制改革扎实有序。行政审批项目精简到195项，全省最少，128项审批项目网上审批，市、县两级审批网络实现了互联互通。大力实施开放带动战略，招商引资165亿元，直接利用境外资金1.17亿美元。培育上市后备企业33家，2家进入证监会审核程序。对外贸易保持较快增长，进出口总额达到8.9亿美元，增长15.3%；其中，出口6.9亿美元，增长12.1%。

（六）高度重视改善民生。着力办好惠及民生的“十大实事”，全面推进“创业富民、就业惠民”工程，城镇新增就业人员8.2万人，下岗失业人员实现再就业1.9万人，农村劳动力新增转移就业14.5万人。对农村中小学生继续实行“两免一补”，免除了城市义务教育阶段学生学杂费，维修改造农村中小学校舍5.2万平方米、新建校舍6.5万平方米。养老、医疗、失业等社会保险覆盖面进一步扩大，农村低保对象补助标准提高到每月50元，新型农村合作医疗参合标准提高到90元，农村“五保户”集中供养率达到45％。全市建设经济适用房8万平方米、廉租住房1.1万平方米。四川灾区对口援建工作有序推进，援建江油市的第一批12个项目全面实施。

（七）积极推进民主法治。坚持依法行政，自觉接受人大及其常委会的监督，支持政协参政议政，办理人大代表议案建议63件、政协委员提案240件。大力推行政务公开，加强电子政务建设，政府网站、市长信箱等民情通道更加通畅。扎实推进“平安许昌”建设，严厉打击各种犯罪，不断完善社会治安防控体系，人民群众安全感明显增强，荣获“河南省平安建设先进省辖市”称号。深入开展安全生产隐患治理，切实强化煤炭生产安全监管，安全生产大局总体平稳。妥善应对问题奶粉事件，全面开展了奶业和食品药品安全专项整治。深入开展源头预防和治理腐败工作，强化效能监察，群众反映强烈的问题得到有效解决。军地军民更加团结，外事、侨务、对台工作全面加强，民族宗教、地方史志、计划生育、档案、统计、地震、气象等工作都取得了新的成绩。

各位代表！

2008年，我们上下一心，奋发有为，在工作中较好地把握了以下几点：一是始终以科学发展观统领经济社会发展全局，着力转变发展方式，不断提高科学发展水平，注重全面协调可持续发展；二是始终以积极进取的态度应对危机，大力推进项目建设、结构调整、企业重组和节能减排等工作，增创跨越发展新优势；三是始终以求真务实的作风推动发展，奋力破解当前发展困局，着力夯实长远发展基础；四是始终以保障和改善民生为重点，加大支出，办好实事，真正让人民群众共享改革发展的成果。所有这些都标志着，过去的一年，我们在危机中迈出了经济社会发展的新步伐，在困难中培育了加快发展的新优势，在考验中续写了富民强市的新篇章。

各位代表！

过去一年的成就来之不易，是省委、省政府和市委审时度势、正确决策的结果，是人大、政协、社会各界和衷共济、全力支持的结果，是全市人民克难攻坚、团结实干的结果。在此，我代表市人民政府，向工作在全市各条战线上的广大干部群众、解放军指战员、武警官兵表示衷心的感谢！向所有关心、支持许昌发展的各界人士表示衷心的感谢！

在肯定成绩的同时，我们也清醒地看到，我市经济社会发展中还存在许多不容忽视的矛盾和问题。受国际金融危机影响，市场有效需求不足日益凸显，企业生产经营困难不断增加，保增长的压力在加大；农村经济发展面临新困难，基础设施依然薄弱，农民持续增收的难度进一步增大；民生欠账仍然较多，就业问题日益突出，安全生产形势更加严峻，社会保障和社会稳定任务十分艰巨；政府职能和作风转变还不到位，一些政府部门大局意识、服务意识还不够强，提升行政效能、优化发展环境任重道远。我们要始终保持清醒头脑，切实增强危机意识和忧患意识，采取积极有效措施，沉着应对，奋发有为，努力做好各项工作，绝不辜负全市人民的期望和重托。

二、2009年工作思路和发展预期

今年是实施“十一五”规划的关键之年，也可能是新世纪以来我市经济发展最为困难的一年，改革发展稳定的任务十分繁重。当前，国际金融危机尚未见底，对实体经济的影响逐步加深，全球经济增长明显衰退，外部因素、短期问题与长期矛盾相互交织，使我市经济下行风险继续加大，面临着严峻挑战。但总体上看，许昌仍处在重要战略机遇期，经济发展的基本态势没有逆转。全球经济深刻调整必将促进国内外产业转移重组，有利于我们扩大开放、借力发展；国家实施积极的财政政策和适度宽松的货币政策，推出一揽子计划，有利于我们加强薄弱环节建设，赢得机遇，加快发展；危机形成的倒逼机制和区域竞争格局重塑，有利于我们加快产业升级、转变发展方式；许昌正处于工业化、城镇化加速发展时期，基础设施建设、产业发展、居民消费等方面空间较大，有利于我们扩内需、保增长；经过改革开放30年的发展，许昌的产业基础更加坚实，体制机制更加灵活，后发优势更加突出，创业氛围更加浓厚，有利于我们应对挑战、跨越发展。我们完全有信心、有条件、有能力克服困难，战胜挑战，推动全市经济社会又好又快发展。

今年政府工作的总体思路是：全面贯彻党的十七大、十七届三中全会、中央经济工作会议、省委八届九次全会和市委五届九次全会精神，以邓小平理论和“三个代表”重要思想为指导，深入贯彻落实科学发展观，把保增长作为首要任务，把扩需求作为根本途径，把调结构作为主攻方向，把加强基础建设作为长远支撑，把深化改革开放作为强大动力，把切实改善民生作为出发点和落脚点，积极应对，奋发有为，化危为机，跨越发展，努力保持经济平稳较快发展，为实现富民强市目标奠定坚实基础。

今年经济和社会发展的主要预期目标是：生产总值增长11%，地方财政一般预算收入增长11%，全社会固定资产投资增长21%，社会消费品零售总额增长15%，实际利用外商直接投资增长12%，外贸出口总额增长12%，居民消费价格涨幅控制在4%以内，城镇居民人均可支配收入实际增长8%，农民人均纯收入实际增长5%，城镇登记失业率控制在4%以内，人口自然增长率控制在6‰以内，万元生产总值能耗下降4%，化学需氧量控制在1.33万吨以内，二氧化硫排放量控制在3.52万吨以内。

做好今年工作，必须做到5个结合：一是坚持增强信心与积极应对相结合。形势越严峻，越要坚定信心，信心比黄金更珍贵。要善于在纷繁复杂的形势中科学把握，始终保持昂扬向上的斗志和干劲，在沉着应对、扎实工作中奋发有为。二是坚持扩大投资与可持续发展相结合。投资拉动既是保增长的应急之举，也是实现可持续发展的根本之策。要抓住中央一揽子促进计划的机遇，加快启动一批有利于可持续发展的大型工程和重要基础设施项目，为经济社会持续健康发展提供战略支撑。三是坚持促进经济增长与转变发展方式相结合。资源、环境的制约是发展的最大隐忧，决不能因经济困难而放松节约能源资源、保护生态环境。要充分利用总量矛盾缓解的机遇，强化节能减排，加强生态环境建设，促进产业结构调整和优化升级。四是坚持扩大内需与改善民生相结合。经济增长的真正和持久动力是最终消费需求，经济发展的根本目的是满足人民群众日益增长的物质文化需要。要把保障和改善民生作为扩大消费、促进增长的有效途径，改善人民生活，增强消费能力，为经济增长提供持久动力。五是坚持加强基础设施建设与统筹城乡发展相结合。加强基础设施建设是统筹城乡发展的关键环节，既要完善城市功能，加快城镇化进程，又要加快农村基础设施建设步伐，缩小城乡差距，促进城乡协调发展。

做好今年工作，必须干在当前，谋在长远，加紧谋划启动事关全局的重大专项：以省委、省政府加快中原城市群建设新的布局为契机，按照产城融合的理念，进一步完善提升许昌至长葛推进区规划建设；全面启动中原电气谷建设，培育壮大战略支撑产业；完善现代城镇体系，围绕“带状城市、组团发展”，逐步推进“一中心五组团”的空间对接；加大文化旅游资源整合力度，大力发展文化旅游产业；着力打造“北方花都”，提升花卉园艺产业发展水平；坚持优先发展教育，继续推进教育资源整合；认真落实服务业发展规划，实现服务业发展新突破；搭建好投融资平台，为重点项目建设提供资金保障；大力发展社会事业，使全市人民共享改革发展成果。

三、把保持经济平稳较快发展作为首要任务

坚持扩内需、保增长与调结构、增效益相统一，加强调控，积极应对，尽快扭转经济增速下滑趋势，努力形成新的经济增长点和竞争优势。

（一）切实增强投资拉动能力。按照“项目建设年”的要求，大力实施“3816”投资促进计划。围绕基础产业、基础设施、基础要素3大基础建设，在工业、农林水利、交通、能源、城镇建设、现代服务业、节能减排和生态建设、社会事业和改善民生8个重点领域，实施100个亿元以上的重大项目，带动全社会固定资产投资600亿元。能源方面，加大泉店煤矿、首山一矿、禹州电厂二期工程、西区热电厂等项目建设力度；工业方面，加快实施帝豪品牌卷烟专线、许继风力发电设备、许昌铁矿等50个重点项目；社会事业方面，加快实施市会展中心、文博馆、体育馆、许昌学院扩建、许昌职业技术学院二期、东城区医院、中心医院外科大楼等民生项目；交通设施方面，积极实施郑漯高速公路拓宽改造许昌段及许昌北出口工程、石武铁路客专许昌段及许昌东站、许禹铁路改造、安信快速通道许昌段、许昌至鄢陵城际快速通道等重大项目；农林水利方面，开工建设南水北调主干线许昌段，大力推进“金三角”烟叶园区、鄢陵县名优花木生产科技园区等项目建设。今明两年，要紧紧抓住中央实施一揽子计划的机遇，抓紧谋划一批重大项目，做好前期，争取对接，力争郑州机场许昌客货枢纽港、禹州电厂三期工程、襄城坑口电厂等重大项目取得新的突破。

（二）竭力为企业排忧解难。按照“企业服务年”的要求，实施“800家企业重点服务计划”，切实解决企业生产经营中存在的突出问题。加强企业信用担保体系建设，推动银企交流合作，帮助企业破解资金瓶颈。认真落实财税、社会保险等优惠政策，全面清理涉企收费和达标评比活动，减轻企业负担。组织好煤电油气运供需衔接，努力保障重点企业要素资源供给。支持企业保存实力，开展促销活动，大力开拓市场；引导企业增强活力，提高管理水平，降低生产成本；帮助企业储备潜力，加强自主创新，优化产品结构，增强发展后劲，使企业在下一轮发展中赢得主动、抢占先机。

（三）强力推进经济结构调整。以建设现代产业体系为目标，大力发展集群经济，推进产业结构优化升级。加快建设“一带十区二十个产业集群”，着力把电力装备、发制品、不锈钢等产业培育成全国知名的产业集群。全面启动中原电气谷核心区建设，加快实施中原电气谷创业服务中心等重点项目。大力实施“千百亿工程”，积极扶持40户重点企业、100户“小巨人”企业和20户重点高新技术企业，促进企业组织结构的调整优化和兼并重组。着力构建自主创新体系，加强许继集团、瑞贝卡公司、青山金汇等重点企业技术改造，实施“品牌兴市”战略，新组建工程技术研究中心10家以上，科技进步对经济增长的贡献率达到53%。加快发展现代服务业，抓好13个投资亿元以上的服务业重点项目建设，完成投资19.4亿元；积极推进众品食业冷链物流基地、河南万里集团物流园区等重点项目，打造区域物流中心；突出抓好禹州文化改革发展试验区、三国文化产业园区建设，推进魏武帝广场等14个文化旅游产业重点项目，完成年度投资7.2亿元；加快建设三鼎丹尼斯百货广场、胖东来国际商城等大型商贸服务业项目，构建区域性中心城市商贸中心。大力发展循环经济，推进低碳经济，严格落实节能减排，实施余热发电、工业点源治理等项目，继续推进污水处理、垃圾处理和电厂脱硫等项目建设。加快建设长葛市大周镇再生金属循环经济产业园、襄城县煤化工循环经济产业园。推动煤炭资源整合，积极创造条件发展煤化工产业园。

（四）加快构建现代城镇体系。大力实施中心城市带动战略，加快许昌至长葛带状城市新区规划建设，着力构建市区、县（市）城区和特色乡镇协调发展的现代城镇体系，城镇化率提高1.8个百分点。扎实推进113个城建重点项目及城中村和旧城区改造项目，积极推进半截河社区、邓庄社区等26个城中村改造项目，完成年度投资39.6亿元。开工建设仓库路、潩水路、灞陵路、汇文路等20个断头路贯通工程，扎实推进八一路中段提升改造、过街天桥、地下通道、管网工程、环卫设施、人行道改造等基础设施项目。继续开展“四城同创”，巩固创卫成果，提升创森水平，实现创模目标，夯实文明城市创建基础。扩大城市管理数字化覆盖范围，提升夜景灯饰化和精细化管理水平。加大违章建筑整治力度，确保城市建设有序推进。加强县（市）城区和中心镇基础设施建设，不断完善城市功能，优化县域产业布局，推动县域经济实现更大规模、更高水平的发展。

四、把解决好“三农”问题作为稳定之基

围绕稳粮、增收、强基础、重民生，强化惠农措施，加大投入力度，优化产业结构，促进农业稳定发展、农村经济繁荣、农民持续增收，加快社会主义新农村建设步伐。

（一）全面落实惠农政策。认真落实中央一号文件精神，粮食最低收购价每公斤平均提高0.055元，较大幅度提高农资综合直补、良种补贴、测土配方施肥补贴和农机具购置补贴等标准。积极实施大豆、油菜子、棉花等农产品临时收储政策，严格落实退耕还林补助，落实生猪、奶牛生产扶持政策。各级财政对农业投入增幅高于经常性收入增幅，大幅度增加对农村基础设施建设和社会事业发展的投入，大幅度提高政府土地出让收益、耕地占用税新增收入用于农业的比例，大幅度增加农村公益性建设项目的投入。做好“家电下乡”、“农机下乡”、“汽车、摩托车下乡”等工作，使农民得到更多的实惠。

（二）大力发展现代农业。以推进国家粮食核心区建设为契机，继续实施粮食高产创建活动，着力提高单产，优化品种结构，力争粮食总产稳定在260万吨。继续推进农业结构调整，大力发展特色农业、生态农业、循环农业和有机农业。加快林业生态许昌建设，完成37.6万亩生态建设任务，花木种植面积达到90万亩。出台花卉园艺产业发展规划，举办好第六届中国花卉产业高峰论坛和第九届中原花木交易博览会。积极发展现代畜牧业，推进规模化、标准化健康养殖，畜牧业产值占农业总产值的比重达到46%。大力发展农业产业化经营，新培育80个农民专业合作组织，重点扶植一批带动能力强的农业龙头企业。

（三）加强农村基础设施建设。切实加强农田水利基础设施建设，新解决20万农村居民饮水安全问题，完成10座中小型水库除险加固任务，新打配机井5143眼，发展节水灌溉面积10万亩，新增有效灌溉面积3万亩、旱涝保收田4万亩。加强农业综合开发，建设高标准农田，改造中低产田7万亩。加强农村民生工程建设，新建和提升农村公路600公里，新增户用沼气6万口，完成18个贫困村的整村推进扶贫任务，帮助1.3万人脱贫。继续抓好“万村千乡市场工程”和“新网工程”建设，农村连锁超市覆盖率达到90%，使农村市场体系更加完善。

（四）加快推进区建设步伐。按照“三个集中、六个一体”的要求，加快实现规划体系基本完善、基础设施基本配套、产业支撑作用明显、生态建设特色鲜明、文化旅游呈现亮点、社区建设全面推进、社会事业基本覆盖的目标。突出抓好推进区147个项目建设，完成年度投资86亿元。大力实施18个社区改造项目，加强道路、电力、污水处理等基础设施建设，加快完善文化教育、医疗卫生、创业就业、社会保障等公共服务体系，让推进区在统筹城乡发展中的示范作用充分发挥。各县（市、区）要因地制宜，选好试点，完善机制，强化措施，加快推进城乡一体化进程。

五、把保障和改善民生作为根本要求

越是困难的时候，越要注重改善民生、促进和谐稳定。把保增长、扩内需同改善民生紧密结合起来，继续办好“十大实事”，着力办成一些社会发展急需、人民群众急盼的事情。

（一）坚持以创业促就业。以创建全国创业型城市为抓手，以打造最佳创业基地为目标，认真落实优惠政策措施，大力推进全民创业。发挥政府投资和重大项目带动就业的作用，提高中小企业、劳动密集型产业和服务业吸纳就业的能力。加大就业援助力度，发放小额担保贷款4500万元。完善落实大学生就业的各项政策，引导他们转变观念，拓宽渠道，到基层创业就业。进一步开发就业岗位，强化技能培训，维护合法权益，促进和稳定农民工就业。全市新增农村劳动力转移就业8万人，新增城镇就业人员5万人，下岗失业人员再就业1.1万人，就业困难人员再就业4000人，动态消除城镇“零就业家庭”。

（二）不断完善保障体系。推进社会保险扩面征缴，企业养老保险新增1.5万人以上，城镇职工基本医疗保险新增1万人。完善城乡居民医疗救助制度，出台失地农民养老保险办法，完善低收入群众基本生活保障政策，城乡低保实现动态管理下的应保尽保，农村“五保”集中供养率达到60%。扩大保障性住房建设规模，建设廉租房3.25万平方米、经济适用房27.5万平方米，切实改善城市低收入家庭住房条件。推进事业单位工资制度改革，落实企业最低工资制度，健全农民工工资支付保障机制，企业退休人员基本养老金人均月增加125元。积极发展社会福利事业和慈善事业。

（三）加强薄弱环节建设。坚持优先发展教育事业，巩固提高义务教育，全面落实农村义务教育经费保障新机制和免除城市义务教育学杂费政策。加强教师队伍建设，积极推进素质教育，让中小学生全面发展。继续做好农村中小学校舍维修改造工作，推进标准化建设。大力实施职业教育攻坚计划，扩大中等职业教育招生规模，积极争取省级示范性职业学校建设项目、薄弱学校改造项目、实训基地建设项目，开工建设许昌技师学院。抓好市、县、乡、村4级卫生服务体系建设，提高城镇居民医保参保率和新农合参合率，让更多的人享有基本医疗卫生服务。落实计划生育奖补政策，综合治理出生人口性别比偏高问题，稳定低生育水平。积极发展公益性文化事业，加快完善公共文化体系。抓好文化信息资源共享工程，力争开播地面数字电视，新增有线电视2.5万户。继续推进体育器材进社区工作，广泛开展全民健身运动，提高竞技体育水平。

（四）维护社会和谐稳定。深入排查调处矛盾纠纷，着力解决信访突出问题。严格落实安全生产责任制，切实加强煤矿、交通运输、烟花爆竹等重点行业的安全监管，坚决遏制重特大事故发生。积极创建食品安全市，深入开展食品药品安全专项整治。扎实推进平安创建活动，加强社会治安综合治理。继续做好丹江口水库移民安置和四川灾区对口援建工作。加强国防和民兵预备役工作，重视和支持工会、共青团、妇联等群众性团体工作，做好民族宗教、地方史志、新闻出版、人事、审计、统计、物价、外事、档案、邮政、地震、气象、人防、对台等各项工作。

六、把深化改革扩大开放作为持久动力

积极推进重点领域和关键环节改革，通过改革创新、扩大开放，增强应对危机的能力，破解发展中的难题，赢得发展新机遇。

（一）深入推进经济体制改革。全面完成国有企业产权制度改革，在更广领域、以更大力度推进企业战略重组和优化兼并，着力培育大企业集团；建立健全现代企业制度，从根本上解决企业历史遗留问题。积极稳妥、分类推进事业单位改革，继续推进科技、教育、社会管理等领域改革。扎实推进列入台账的58家企事业单位改革改制工作。深化财税体制改革，实施好增值税、营业税、消费税暂行条例及燃油税费改革方案。继续深化农业银行和农村信用社改革，推进村镇银行建设，积极争取交通银行等境内外金融机构在我市设立分支机构。致力打造诚信许昌，创建中国金融生态城市。大力发展民营经济，推动民营企业加快技术创新，增强经济发展活力。

（二）切实加强政府管理创新。着力抓好政府机构改革，力争上半年完成市级政府机构改革，年底前完成县级政府机构改革。深化行政审批制度改革，抓好市、县、乡3级网络平台建设，加快建设行政审批电子监察系统，不断提高行政审批效率。加强部门预算管理，优化支出结构，逐步建立有利于科学发展的财政导向机制。整合国有资源、资产、资金、资本，搭建投融资平台，提高融资和偿债能力。深化医疗卫生体制改革，促进基本公共卫生服务均等化。积极推进农村服务体系、土地流转等方面的改革，全面完成集体林权制度改革任务。

（三）大力实施开放带动主战略。创新招商引资方式，注重产业招商、园区招商、项目招商、环境招商，利用多种形式，走出去、请进来，积极对接、主动承接国内外产业转移，努力引进一批知名企业集团。大力发展会展经济，举办好三国文化周、钧瓷文化节等活动。积极推进企业上市，引导已上市企业扩大融资。落实好外贸扶持政策，调整优化出口结构，稳定国际市场份额。加强与中西部地区的合作交流，深化资源开发与产业发展合作。注重与沿海发达地区建立产业转移联盟，设立产业转移承接园区，吸纳相关产业向我市集群转移。继续推进与郑州的产业对接、空间对接，使郑许呼应发展迈出新的步伐。

七、把政府自身建设作为重要保障

积极推进政府自身建设，不断提高依法行政、为民执政、高效施政、廉洁从政、务实理政的能力和水平，为保增长、保民生、保稳定提供有力保障。

（一）以法治为上，更加注重依法行政。严格执行人大及其常委会的决议、决定，自觉接受人大及其常委会的监督。自觉接受人民政协的民主监督。广泛听取各民主党派、工商联和无党派人士的意见和建议，主动接受社会舆论和人民群众的监督。认真办理人大代表建议和政协提案。规范办事程序和行为准则，坚持用制度管权、按制度办事、靠制度管人。落实重大事项集体决策制度，加强和改进行政复议工作，规范行政执法行为，切实推进严格执法、公正执法、文明执法。

（二）以服务为重，更加注重为民执政。加快转变政府职能，切实提高服务水平。完善为民服务快速通道，优化公共资源配置，为农民和城镇低收入者提供更多更好的公共服务。继续推进社区建设，不断完善社区功能。强化监管，改善公用事业服务。完善突发公共事件应急预案，提高预防和处置能力。加强电子政务建设，深入推进政务公开、村务公开、厂务公开。畅通政府网站、市长信箱、电话信件、110应急联动系统等诉求渠道，及时掌握和解决群众急难盼怨的问题，赢得人民群众的理解、支持和参与。

（三）以效能为先，更加注重高效施政。扎实推进“五个坚持、两转两提”，创新政府工作人员激励约束机制，大力表彰“人民满意的公务员”，不断提升公务员队伍整体素质。深化机关标准化管理，完善目标管理、绩效考评、新闻发布等工作机制，提升行政效能。加强督查问责，完善重点领域和薄弱环节效能过错责任追究办法，严肃查处行政不作为、乱作为、慢作为，确保政令畅通。

（四）以求实为要，更加注重务实理政。坚持问政于民、问需于民、问计于民，多办顺民心、惠民众的事情，多做打基础、管长远的工作。坚持科学民主决策，一切从实际出发，自觉按照客观规律办事，力戒决策失误。按照“严、细、深、实、快”的要求，大力精简会议、文件，切实改进会风、文风，着力形成清新、简约、责实、务本的良好政风。

（五）以廉政为本，更加注重廉洁从政。自觉遵守党纪国法，端正行风政风。加强廉政建设，严格落实财政投资评审、财务收支、政府采购等管理规定。建立完善公共资源交易制度，规范交易市场秩序。加强对预算执行、专项资金、重点项目、教育收费、医药购销、农民负担等方面的审计。严肃查办商业贿赂以及严重损害群众利益的案件，依法惩处腐败分子。各级政府都要厉行节约，艰苦奋斗，压减行政费用，和人民群众一道共克时艰。

各位代表！

做好今年的工作，意义重大深远，任务艰巨繁重，形势催人奋进。让我们在中共许昌市委的正确领导下，坚定必胜信心，勇于面对挑战，解放思想，团结拼搏，锐意进取，扎实工作，奋力促进全市经济社会又好又快发展，以优异成绩迎接新中国成立60周年！
